# In utero exposure to mercury and childhood overweight or obesity: counteracting effect of maternal folate status

**DOI:** 10.1186/s12916-019-1442-2

**Published:** 2019-11-28

**Authors:** Guoying Wang, Jessica DiBari, Eric Bind, Andrew M. Steffens, Jhindan Mukherjee, Tami R. Bartell, David C. Bellinger, Xiumei Hong, Yuelong Ji, Mei-Cheng Wang, Marsha Wills-Karp, Tina L. Cheng, Xiaobin Wang

**Affiliations:** 1 Department of Population, Family and Reproductive Health, Center on the Early Life Origins of Disease, Johns Hopkins Bloomberg School of Public Health, 615 N. Wolfe Street, Baltimore, MD 21205-2179 USA; 2 Division of Research, Office of Epidemiology and Research, Maternal and Child Health Bureau, Health Resources and Services Administration, 5600 Fishers Ln, Rockville, MD 20852 USA; 3 Metals Laboratory, Environmental and Chemical Laboratory Services, The New Jersey Department of Health, Trenton, NJ 08628 USA; 4 Mary Ann & J. Milburn Smith Child Health Research, Outreach and Advocacy Center, Stanley Manne Children’s Research Institute, Ann & Robert H Lurie Children’s Hospital of Chicago, 2430 N Halsted St, Chicago, IL 60614 USA; 5 Department of Neurology, Boston Children’s Hospital, Harvard Medical School, 300 Longwood Ave, Boston, MA 02115 USA; 6 Department of Biostatistics, Johns Hopkins Bloomberg School of Public Health, 615 N. Wolfe street, Baltimore, MD 21205 USA; 7 Department of Environmental Health and Engineering, Johns Hopkins Bloomberg School of Public Health, 615 N. Wolfe street, Baltimore, MD 21205 USA; 8 Department of Pediatrics, Johns Hopkins School of Medicine, 615 N. Wolfe street, Baltimore, MD 21205 USA

**Keywords:** Diabetes, Folate, In utero, Mercury, Metal, Nutrient, Overweight, Obesity

## Abstract

**Background:**

Low-dose mercury (Hg) exposure has been associated with cardiovascular diseases, diabetes, and obesity in adults, but it is unknown the metabolic consequence of in utero Hg exposure. This study aimed to investigate the association between in utero Hg exposure and child overweight or obesity (OWO) and to explore if adequate maternal folate can mitigate Hg toxicity.

**Methods:**

This prospective study included 1442 mother-child pairs recruited at birth and followed up to age 15 years. Maternal Hg in red blood cells and plasma folate levels were measured in samples collected 1–3 days after delivery (a proxy for third trimester exposure). Adequate folate was defined as plasma folate ≥ 20.4 nmol/L. Childhood OWO was defined as body mass index ≥ 85% percentile for age and sex.

**Results:**

The median (interquartile range) of maternal Hg levels were 2.11 (1.04–3.70) μg/L. Geometric mean (95% CI) of maternal folate levels were 31.1 (30.1–32.1) nmol/L. Maternal Hg levels were positively associated with child OWO from age 2–15 years, independent of maternal pre-pregnancy OWO, diabetes, and other covariates. The relative risk (RR = 1.24, 95% CI 1.05–1.47) of child OWO associated with the highest quartile of Hg exposure was 24% higher than those with the lowest quartile. Maternal pre-pregnancy OWO and/or diabetes additively enhanced Hg toxicity. The highest risk of child OWO was found among children of OWO and diabetic mothers in the top Hg quartile (RR = 2.06; 95% CI 1.56–2.71) compared to their counterparts. Furthermore, adequate maternal folate status mitigated Hg toxicity. Given top quartile Hg exposure, adequate maternal folate was associated with a 34% reduction in child OWO risk (RR = 0.66, 95% CI 0.51–0.85) as compared with insufficient maternal folate. There was a suggestive interaction between maternal Hg and folate levels on child OWO risk (*p* for interaction = 0.086).

**Conclusions:**

In this US urban, multi-ethnic population, elevated in utero Hg exposure was associated with a higher risk of OWO in childhood, and such risk was enhanced by maternal OWO and/or diabetes and reduced by adequate maternal folate. These findings underscore the need to screen for Hg and to optimize maternal folate status, especially among mothers with OWO and/or diabetes.

## Background

Mercury (Hg) is a persistent and widespread environmental pollutant and is highly toxic to human health worldwide. Exposure to toxic Hg is ubiquitous in the general US population [1]. The National Health and Nutrition Examination Survey found that 85.2% of the population had detectable levels of Hg [1]. Methylmercury (MeHg, the major constituent of organic Hg) is of particular concern due to its ability to cross the placenta and blood-brain barrier during pregnancy, as well as bioaccumulation [2]. Neuro-toxic effects of MeHg have been well studied [3], and MeHg’s role in cardiometabolic health is beginning to be recognized [4, 5]. Studies in adults have found that low-dose Hg exposure was associated with obesity [6] and visceral adipose tissue [7], suggesting that Hg is an obesogen.

The developing fetus is particularly vulnerable to nutritional and environmental exposures [8, 9]. Previous studies suggest that obesity epidemic could be, in part, due to in utero exposures to adverse environments [10, 11]. Although Hg has been recognized as an obesogen [6, 12, 13], literature is limited for the link of in utero Hg exposure and obesity in later life. Especially, the role of Hg in the inter-generational risk of obesity is yet to be explored. Given childhood obesity persists into adulthood and is linked to cardiovascular disease in later life, a critical unanswered question is whether in utero low-dose Hg exposure increases a child’s risk for obesity in childhood and beyond.

In light of widespread maternal exposure to Hg in the USA, there is an urgent need to identify potentially important mitigating factors. Because antioxidants are desirable in the process of Hg detoxification [14], folate, an essential B vitamin, may be a candidate due to its antioxidant and anti-inflammatory properties [15, 16]. Studies have demonstrated that folate has protective effects on cardiovascular diseases [17] and might reduce the adverse impacts of environmental chemicals on the fetus [18]. One study has documented an inverse relationship between serum folate levels and blood Hg concentration during pregnancy [19]. Furthermore, our previous studies unveiled a protective effect of folate on inter-generational risks of obesity and elevated blood pressure [11, 20]. However, the role of folate in the setting of in utero Hg exposure and inter-generational link of obesity is yet to be examined.

Using a well-established, population-based birth cohort, this study sought to characterize the potential adverse effect of in utero Hg exposure on childhood overweight or obesity (OWO) up to 15 years of age. We also assessed whether maternal pre-pregnancy OWO and/or diabetes enhances the toxic effect of in utero Hg exposure. In addition, we evaluated to what extent adequate maternal folate status during pregnancy counteracts the toxicity of in utero Hg exposure on childhood OWO.

## Study population and methods

### Study population

The study protocol was approved by the Institutional Review Boards of Boston Medical Center and Johns Hopkins Bloomberg School of Public Health. Written informed consent was obtained from each study child’s biological mother or the study child. Participants of this study came from the Boston Birth Cohort (BBC), who were enrolled at birth and followed prospectively thereafter at the Boston Medical Center. Detailed information on participant enrollment has been described previously [21]. Briefly, the mother-infant pairs were enrolled 24 to 72 h after delivery. After obtaining written informed consent, trained research staff interviewed mothers using a standardized questionnaire. Maternal blood was drawn for measurement of Hg in red blood cell (RBC) and plasma folate concentrations. Pertinent clinical information was obtained by a review of maternal and infant medical records, including prenatal ultrasonographic reports, laboratory reports, and information on pregnancy complications, and birth outcomes. As of July 2018, 3163 children who enrolled in the BBC and received primary care at the Boston Medical Center were followed. Of these children, 1551 mothers had measurements taken for Hg concentration in the RBCs. There were 109 children excluded due to a lack of body mass index (BMI) data between ages 2–15 years. Finally, this study included 1442 mother-child pairs who had complete data (Additional file 1: Figure S1). The characteristics were similar between the included and excluded samples, except that more Black women and less preterm births were in the included samples (Additional file 1: Table S1).

### Ascertainment of maternal Hg levels in RBCs

Maternal RBC-Hg concentrations were measured using inductively coupled plasma mass spectrometry (ICP-MS) on an Agilent 8900 QQQ (Agilent Technologies Inc., Santa Clara, CA) at the New Jersey Department of Health, Environmental and Chemical Laboratory Services, Metals Laboratory, Trenton, New Jersey. This laboratory is certified by the US Centers for Disease Control and Prevention, Clinical Laboratory Improvement Amendment, and Clinical Laboratory Improvement Services for analysis of clinical samples. The analytical procedures followed standard protocols for quality control and assurance. The quality control includes a calibration curve comprised of a minimum of five points, continuous calibration verification, continuous calibration blank verification, duplicate samples, laboratory fortified matrix samples, blind duplicates, and a minimum of four second source quality control samples for every run. Calibration stock solution (catalog# SM-2107-042-50, High-Purity Standards, SC) and second source quality control samples (Blood Metals Quality Control Specimens, Lot 018, Wisconsin State Laboratory of Hygiene, WI) were used as National Institute of Standards and Technology (NIST)-traceable certified reference materials to calibrate and monitor the accuracy and interday and intraday repeatability of the analysis. The second source samples must recover within the established recovery range set by the manufacturer or within a tighter range established by the laboratory. The coefficient of variation (CV) was < 5.0%. The limit of detection (LOD) was 0.28 μg/L. If the concentration was below the LOD, the value was assigned to be the value of the LOD divided by the square root of two.

### Ascertainment of maternal plasma folate concentrations

Maternal plasma folate concentrations were measured by a commercial laboratory via chemiluminescent immunoassay using a MAGLUMI 2000 Analyzer (Snibe Co., Ltd., Shenzhen, China). Each run included a calibration curve with five points and three internal quality controls. If the value of folate was above the maximum LOD, plasma samples were diluted at a ratio of 1:2 and then re-run. The inter-assay CV was < 4% [22]. In our previous study [11], we found that maternal plasma folate level ≥ 20.4 nmol/L was associated with a lower risk of child OWO and mitigated the inter-generational link of obesity. As such, we defined adequate maternal folate as maternal plasma folate ≥ 20.4 nmol/L.

### Definition of maternal characteristics

Maternal variables, including age at time of delivery, race/ethnicity, parity, education, pre-pregnancy height and weight, cigarette smoking, and fish consumption, were based on maternal questionnaire interview. Maternal pre-pregnancy BMI was calculated as pre-pregnancy weight (kg) divided by squared height (m), and further dichotomized into non-OWO (BMI < 25 kg/m^2^) and OWO (BMI ≥ 25 kg/m^2^). Fish consumption was grouped into none, 1–2 servings/week, and ≥ 3 servings/week. Maternal pregnancy complications, including diabetes mellitus (either gestational diabetes or pre-existing diabetes), hypertensive disorders (pre-eclampsia, eclampsia, gestational hypertension, chronic hypertension, and HELLP [hemolysis, elevated liver enzymes, and low platelets syndrome]), were abstracted from medical records. Gestational age was estimated based on the first day of the last menstrual period, as recorded in the maternal medical record, or early (< 20 weeks) prenatal ultrasonographic results, as detailed in a previous publication [21].

### Assessment of child’s birth outcomes and breastfeeding status

Child’s birthweight and gender were abstracted from the medical records*.* Birthweight for gestational age was calculated according to an established local reference population, and controlled for infant gender, gestational age, and ethnicity [21]. Fetal growth pattern was defined by birthweight for gestational age and grouped into small for gestational age (SGA) (gestational age specific birthweight < 10th percentile), large for gestational age (LGA) (birthweight > 90th percentile), and appropriate for gestational age (AGA) (birthweight in the 10th to 90th percentile for gestational age). Information regarding infant breastfeeding history was primarily assessed within the first 2 years of follow-up visits, and grouped into exclusively breastfeeding, exclusively formula feeding, or mixed breast and formula feeding [23].

### Child BMI and overweight or obesity in childhood

Child weight and height were extracted from the medical records, which were measured by trained medical staff using the same clinical protocol and equipment during well-child visits. Before data analyses, careful data checking and cleaning of weight and height data was performed. At first, we removed extreme or biologically implausible values. Then, we identified outliners or erroneous height and weight values based on growth curve. When possible, erroneous height and weight values were corrected, otherwise the points were deleted. Age- and sex-specific BMI *z*-scores and percentiles were calculated using US national reference data [24], derived from a US representative sample. OWO was defined as BMI ≥ 85th percentile for age and sex [25]. The BBC has used rolling enrollment, so the length of postnatal follow-up and number of well-child visits varied greatly across study participants. It is known that OWO at an older age is more likely to persist into adulthood. Thus, we chose the last visit in each time period as the end point for OWO.

### Statistical analysis

Demographic and clinical data are presented as either mean ± standard deviation (SD) or *n*(%) stratified by maternal Hg categories. Unadjusted trend *p* values across maternal Hg categories were calculated by Mantel-Haenszel *χ*^2^ for categorical variables and linear regression for continuous variables. The relationship between maternal Hg and child OWO is displayed using locally weighted regression smoothing plots (implemented using PROC LOESS in SAS, a nonparametric regression method).

Relative risk (RR) was estimated by Poisson regression with robust error variance to determine an association between maternal Hg and child OWO, adjusting for important covariates, including maternal age, race/ethnicity, education, parity, smoking, pre-pregnancy OWO, diabetes, hypertensive disorders, preterm birth, fetal growth pattern, and child’s breastfeeding status. To examine the persistence of the association from preschool age to school age to adolescents, we performed similar analyses stratified by child age at outcome assessment (e.g., at age 2–5 years, 6–9 years, and 10–15 years). In addition, we evaluated the joint risk attributed to maternal Hg categories and either or both pre-pregnancy OWO and diabetes. We tested the interaction of maternal pre-pregnancy OWO (as a binary variable) and Hg levels (as a continuous variable) or the interaction of maternal diabetes status and Hg levels on child’s risk of OWO by adding a multiplicative term in the models. Effect modification was assessed by the likelihood ratio test using an a priori *α* value of 0.05. We used same methods to investigate whether maternal adequate folate status can ameliorate the adverse effect of maternal Hg on child risk of OWO.

Finally, to examine the robustness of the results and biological plausibility, we conducted a series of sensitivity analyses: subgroup analyses, including among fish consumers, term births, Black children, and breastfed children, and sequential models adding more covariables of interest. We also performed similar analyses stratified by child’s sex. All *p* values were from two-sided tests, and all statistical analyses were performed using SAS v.9.4 (SAS Institute Inc., Cary, NC, USA).

## Results

Our study population consisted of 1442 mother-child pairs of which 722 (50·1%) children were boys and 967(67·1%) were Black. The age range of children at his/her last visit was 2–15 years. Hg was detected in 89% of mothers. The median [interquartile range (IQR)] of maternal RBC-Hg was 2.11 (1.04–3.70) μg/L. The distribution of maternal RBC-Hg is presented according to race/ethnicity and maternal OWO/diabetes status (Additional file 1: Figure S2). Geometric mean [95% confidence interval (CI)] of maternal folate plasma was 31.1 (30.1–32.1) nmol/L. In all, 21.4% of mothers had plasma folate levels < 20.4 nmol/L. Maternal and child characteristics stratified by maternal Hg quartiles are presented in Table 1. Older, non-smoking, Black, and multiparous mothers had higher Hg levels. A higher frequency of fish intake was associated with higher Hg levels. Lower plasma folate levels were associated with increasing Hg levels. Children of mothers with higher Hg levels tended to be female, older, and breastfed.

**Table 1:** The characteristics of the study population (*n* = 1442)

	Maternal RBC mercury level (μg/L) in quartiles				
	Q1 0·39–1·04	Q2 1·02–2·10	Q3 2·12–3·68	Q4 3·70–27·8	P trend
Maternal characteristics					
*n*	360	361	359	362	
Age, years	26·8 ± 6·5	28·0 ± 6·0	29·1 ± 6·7	30·3 ± 6·3	< 0·001
Race					< 0·001
Black	192 (53·3)	225 (62·3)	265 (73·8)	285 (78·7)	
Non-black	168 (46·7)	136 (37·7)	94 (26·2)	77 (21·3)	
Education					0·367
High school and less	234 (65·0)	243 (67·3)	233 (64·9)	226 (62·4)	
Beyond high school	126 (35·0)	118 (32·7)	126 (35·1)	136 (37·6)	
Smoking					< 0·001
Not smoker	279 (77·5)	284 (78·7)	305 (85·0)	324 (89·5)	
Smoker	81 (22·5)	77 (21·3)	54 (15·0)	38 (10·5)	
Parity					0·001
Nulliparous	177 (49·2)	150 (41·6)	139 (38·7)	136 (37·6)	
Multiparous	183 (50·8)	211 (58·4)	220 (61·3)	226 (62·4)	
Pre-pregnancy BMI, kg/m^2^	26·4 ± 6·8	27·2 ± 7·6	27·2 ± 6·7	26·5 ± 5·7	0·769
Overweight or obesity	176 (48·9)	185 (51·2)	203 (56·6)	197 (54·4)	0·063
Diabetes	44 (12·2)	45 (12·5)	45 (12·5)	50 (13·8)	0·537
Hypertensive disorder	45 (12·5)	50 (13·9)	61 (17·0)	60 (16·6)	0·068
Fish intake (serving/week)					< 0·001
0	163 (45·3)	80 (22·2)	55 (15·3)	35 (9·7)	
1–2	183 (50·8)	253 (70·1)	246 (68·5)	239 (66·0)	
≥ 3	14 (3·9)	28 (7·7)	58 (16·2)	88 (24·3)	
Plasma folate (nmol/L)*	34·6 (32·5–36·8)	30·1 (28·3–32·1)	29·4 (27·5–31·3)	30·7 (28·8–32·7)	< 0·001
Child’s characteristics					
Age, years	7·6 ± 2·9	8·0 ± 3·2	8·6 ± 3·3	8·4 ± 3·0	0·001
Gender					0·002
Boy	196 (54·4)	190 (52·6)	180 (50·1)	156 (43·1)	
Girl	164 (45·6)	171 (47·4)	179 (49·9)	206 (56·9)	
Birthweight, g	2982 ± 773	2955 ± 776	2972 ± 790	3015 ± 818	0·775
Gestational age, weeks	38·0 ± 3·2	37·9 ± 3·3	37·8 ± 3·2	38·1 ± 3·1	0·998
Preterm birth	88 (24·4)	90 (24·9)	85 (23·7)	85 (23·5)	0·681
Fetal growth					0·176
AGA	290 (80·6)	286 (79·2)	279 (77·7)	281 (77·6)	
SGA	38 (10·5)	37 (10·3)	39 (10·9)	37 (10·2)	
LGA	32 (8·9)	38 (10·5)	41 (11·4)	44 (12·2)	
Breastfeeding					0·013
Formula only	90 (25·0)	105 (29·1)	88 (24·5)	65 (17·9)	
Breastfeed exclusively	28 (7·8)	31 (8·6)	21 (5·9)	39 (10·8)	
Both	242 (67·2)	225 (62·3)	250 (69·6)	258 (71·3)	
Overweight or obesity	138 (38·3)	154 (42·7)	146 (40·7)	174 (48·1)	0·019
Number of BMI measures	3·8 ± 2·3	4·0 ± 2·5	4·1 ± 2·6	4·0 ± 2·4	< 0·001

*Geometric mean (95% CI); *Q* quartile, *RBC* red blood cell, *AGA* appropriate for gestational age, *SGA* small for gestational age, *LGA* large for gestational age

### Maternal RBC-Hg levels and child OWO

**Fig. 1:**
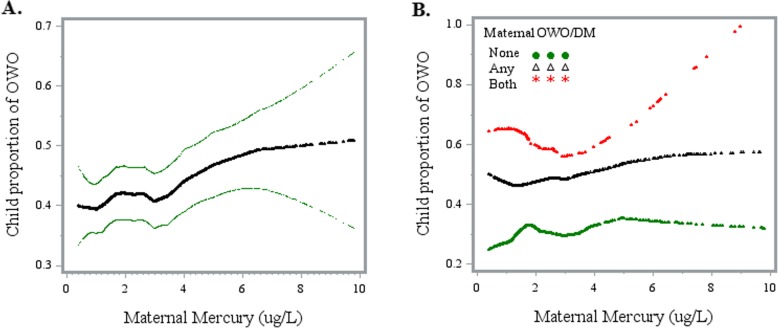
Association between maternal RBC-Hg concentrations and offspring overweight or obesity. Abbreviation: OWO, overweight or obesity; DM, diabetes. Panel **a** displays the crude association between maternal RBC-Hg concentration and offspring proportion of OWO. Panel **b** displays the association stratified by maternal OWO/DM condition. None, non-OWO and non-diabetic; Any, either OWO or diabetes; Both, OWO and diabetes

As shown in Fig. 1, maternal Hg levels were positively associated with an increased risk of child OWO (panel a). When stratified by maternal pre-pregnancy OWO and/or diabetes, the association was strongest among OWO and diabetic mothers (panel b). Furthermore, the risk of child OWO among OWO and diabetic mothers was the highest across the whole Hg spectrum. Multivariate regression models (Table 2) showed that the increased risk of child OWO was mainly concentrated in the top Hg quartile (Q4) with an RR of 1.24 (95% CI 1.05–1.47), while second (Q2) and third (Q3) Hg quartiles were not significantly associated with the risk of child OWO, as compared with the lowest quartile (Q1). When we combined Q1–Q3 as the reference, the top Hg quartile was associated with increased risk of child OWO (RR = 1·19; 95% CI 1.05–1.35; *p* = 0·007). These associations persisted from preschool age (2–5 years), to school age (6–9 years) and into early adolescence (10–15 years).

**Table 2:** Association of maternal mercury levels with child risk of overweight or obesity

Age group	Mercury quartile			Crude			Adjusted		
		*n*	Case, *n*(%)	RR	95% CI	*p*	RR	95% CI	*p*
Total sample 2–15 years (*n* = 1442)									
Q1		360	138 (38·3)	1·00			1·00		
Q2		361	154 (42·7)	1·11	0·93–1·33	0·238	1·09	0·92–1·30	0·313
Q3		359	146 (40·7)	1·06	0·89–1·27	0·522	1·03	0·86–1·23	0·748
Q4		362	174 (48·1)	1·25	1·06–1·49	0·009	1·24	1·05–1·47	0·013
*P* trend						0·047			0·098
Q1–Q3		1080	438 (40·6)	1·00			1·00		
Q4		362	174 (48·1)	1·19	1·04–1·35	0·010	1·19	1·05–1·35	0·007
2–5 years (*n* = 1395)									
Q1		346	113 (32·7)	1·00			1·00		
Q2		347	139 (40·1)	1·23	1·01–1·50	0·044	1·20	0·99–1·45	0·061
Q3		347	138 (39·8)	1·22	1·00–1·49	0·053	1·15	0·95–1·41	0·160
Q4		355	159 (44·8)	1·37	1·13–1·66	0·001	1·33	1·10–1·61	0·003
*P* trend						0·015			0·067
Q1–Q3		1040	390 (37·5)	1·00			1·00		
Q4		355	159 (44·8)	1·19	1·04–1·37	0·013	1·19	1·03–1·36	0·016
6–9 years (*n* = 1030)									
Q1		250	100 (40·0)	1·00			1·00		
Q2		255	118 (46·3)	1·16	0·95–1·41	0·156	1·15	0·94–1·39	0·171
Q3		258	116 (45·0)	1·12	0·92–1·38	0·259	1·09	0·89–1·34	0·399
Q4		267	142 (53·2)	1·33	1·10–1·61	0·003	1·30	1·07–1·58	0·007
*P* trend						0·028			0·100
Q1–Q3		763	334 (43·8)	1·00			1·00		
Q4		267	142 (53·2)	1·21	1·06–1·40	0·006	1·20	1·04–1·38	0·010
10–15 years (*n* = 449)									
Q1		91	38 (41·8)	1·00			1·00		
Q2		106	47 (44·3)	1·06	0·77–1·47	0·716	1·14	0·84–1·56	0·383
Q3		138	67 (48·6)	1·16	0·86–1·57	0·320	1·22	0·91–1·64	0·184
Q4		114	62 (54·4)	1·30	0·97–1·75	0·079	1·40	1·04–1·88	0·028
*P* trend						0·057			0·058
Q1–Q3		335	152 (45·4)	1·00			1·00		
Q4		114	62 (54·4)	1·20	0·98–1·47	0·083	1·22	0·99–1·50	0·061

*Q* quartile, *RR* relative risk, *CI* conference interval. Mercury quartile range: Q1 0.39–1.04 μg/L; Q2 1.04–2.10 μg/L; Q3 2.12–3.68 μg/L; Q4 3.70–27.8 μg/L.

Adjusted for maternal age, race, smoking, education, parity, pre-pregnancy overweight or obesity, diabetes, hypertensive disorder, preterm birth, fetal growth pattern, and breastfeeding status

### Combined effects of maternal pre-pregnancy OWO/diabetes and Hg levels on child OWO

**Fig. 2:**
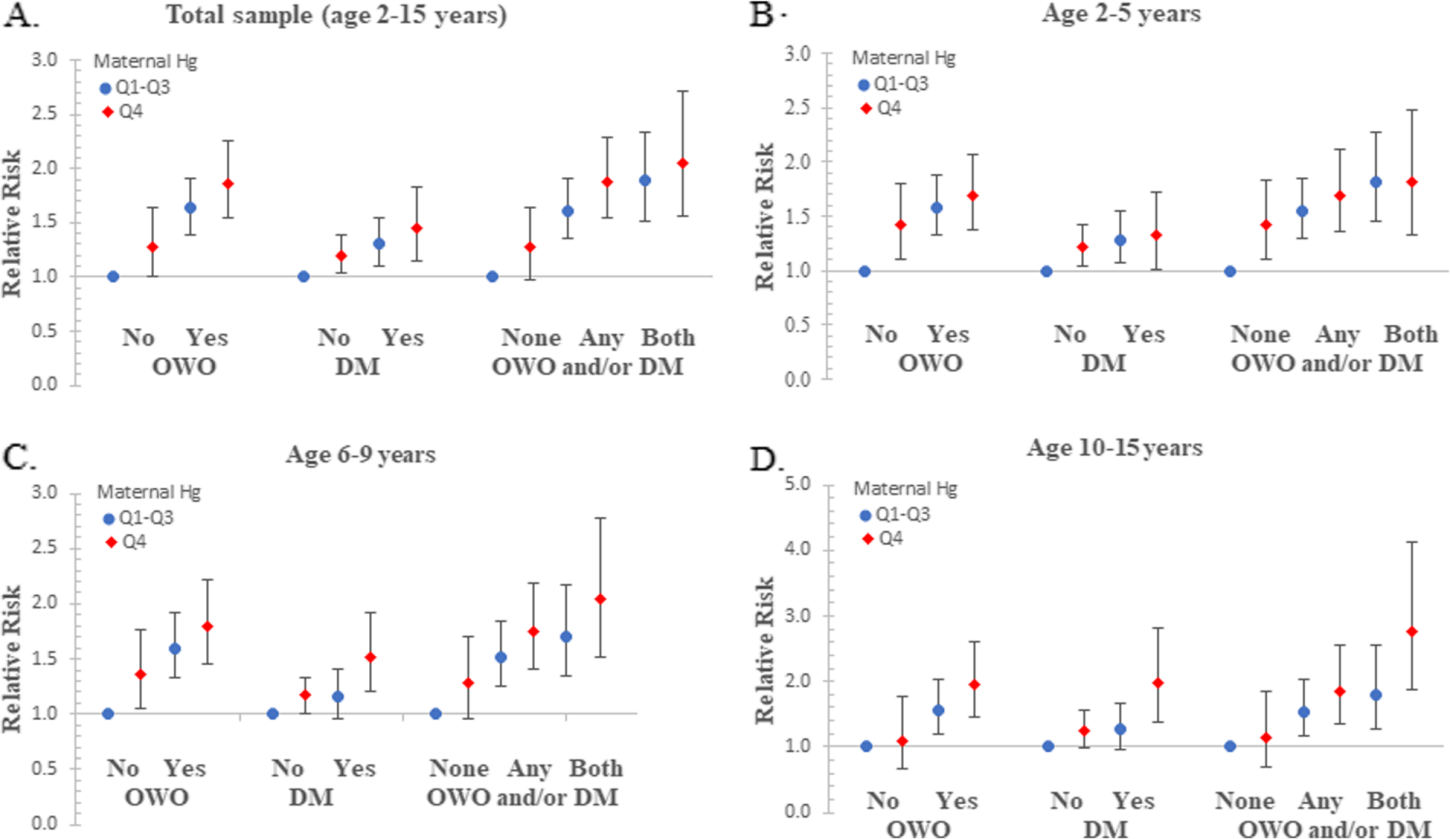
Combined effect of maternal OWO and/or DM and RBC-Hg on the risk of child OWO. OWO, overweight or obesity; DM, diabetes; Q, quartile; The presence of maternal OWO and diabetes was categorized into three groups: none (without OWO and diabetes), any (either OWO or diabetes), and both (OWO and diabetes). Panel **a** displays the associations among total sample at age from 2-15 years; panel **b** displays the associations among children at age from 2-5 years; panel **c** displays the associations among children at age from 6-9 years; panel **d** displays the associations among children at age from 10-15 years

We found a significant combined effect of maternal Hg levels and either or both pre-pregnancy OWO and diabetes on child OWO risk. Children of OWO mothers in the top quartile of Hg had an increased risk of OWO (RR = 1.87; 95% CI 1.55–2.25) compared to those with non-OWO mothers in lower quartiles of Hg (Fig. 2, a). Similarly, the risk of OWO was significantly higher in children of diabetic mothers in the top quartile of Hg than those with non-diabetic mothers in lower quartiles of Hg (RR = 1.45; 95% CI 1·15–1.83). When the presence of maternal OWO and diabetes was categorized into three groups: none (without OWO and diabetes), any (either OWO or diabetes), and both (OWO and diabetes), the risk of OWO (RR = 2.06, 95% CI 1.56–2.71) was highest in children born to mothers with both OWO and diabetes and in the top quartile of Hg, compared with children of mothers without OWO and diabetes and in low quartiles of Hg (Fig. 2a). However, there was no evidence of an interaction between maternal RBC-Hg and pre-pregnancy OWO or between maternal Hg and diabetes with respect to the risk of child OWO (*p* > 0.05). These association patterns were similar for preschool age children (Fig. 2b), school age children (c), and adolescents (d).

### Counteracting effects of maternal folate status against RBC-Hg

Maternal plasma folate was inversely related with Hg concentration (Additional file 1: Figure S3). As shown in Table 3, the rate of child OWO was significantly lower in children whose mothers in the highest Hg quartile and with adequate maternal folate than children whose mothers with a low folate levels (43.6% vs. 60.9%; RR = 0·66; 95% CI 0.51–0·85; *p* = 0.001). Adequate maternal folate status was not associated with reduced child OWO risk (RR = 0.96; 95% CI 0.80–1.15; *p* = 0.658) among children of mothers with Hg levels in the lower three quartiles. The interaction between maternal RBC-Hg and plasma folate on child OWO risk was marginal (*p* = 0.086 for interaction).

**Table 3:** Modifying effects of maternal folate levels with mercury levels on child overweight or obesity risk

Mercury quantile	Maternal folate			Crude			Adjusted		
		*n*	Case, *n*(%)	RR	95% CI	*p*	RR	95% CI	*p*
Q4	Low	64	39 (60.9)	1.00			1.00		
	Adequate	236	103 (43.6)	0.72	0.56–0.91	0.007	0.66	0.51–0.85	0.001
Q1-Q3	Low	195	85 (43.6)	1.00			1.00		
	Adequate	714	289 (40.5)	0.93	0.77–1.11	0.427	0.96	0.80–1.15	0.658
*P* for interaction						0.095			0.086

*Q* quartile. Mercury quartile range: Q1 0.39–3.68 μg/L; Q4 3.70–27.8 μg/L. Adequate folate was defined as plasma folate ≥ 20.4 nmol/L; low folate was defined as plasma folate < 20.4 nmol/L

Adjusted for maternal race, smoking, education, parity, pre-pregnancy overweight or obesity, diabetes, hypertensive disorder, preterm birth, fetal growth pattern, and breastfeeding status

### Subgroup and sensitivity analyses

To control for other potential confounders, we further adjusted for frequency of maternal fish consumption (Additional file 1: Table S2), maternal RBC-selenium levels (Additional file 1: Table S3), and maternal RBC-lead levels (Additional file 1: Table S4). The results were similar to those reported above. A series of subgroup analyses were performed, and the associations were not changed materially among fish consumers (Additional file 1: Table S5), term births (Additional file 1: Table S6), Blacks (Additional file 1: Table S7), boys (Additional file 1: Table S8), girls (Additional file 1: Table S9), and breastfed children (Additional file 1: Table S10). To further evaluate the associations, we analyzed child BMI *z*-score as a continuous outcome (Additional file 1: Table S11), and again the results were consistent.

## Discussion

To the best of our knowledge, this is the first study to investigate in utero Hg exposure and OWO in childhood using a prospective, longitudinal birth cohort. This study provided several new findings. Maternal RBC-Hg levels were positively associated with the risk of child OWO. The highest risk of child OWO was seen among children of mothers with Hg levels in the top quartile. The associations were consistent from preschool age to school age to adolescence. The Hg-related risk of child OWO was further enhanced by maternal pre-pregnancy OWO and/or diabetes but mitigated by adequate maternal folate status. These data suggest that in utero Hg exposure has a long-term consequence on child metabolic health, and optimal folate in utero may act to reduce Hg toxicity. Our findings underscore the need for screening for blood Hg and folate levels in pregnant women, particularly in OWO and/or diabetic women.

Hg is well-known to be a neurotoxicant and is beginning to be recognized as an obesogen [6, 12, 13]. Studies in adults have suggested that blood Hg levels were associated with obesity [6] and visceral adipose tissue (measured by dual-energy *X*-ray absorptiometry) [7]. Measures of Hg in human hair were also associated with BMI [13] and obesity [12]. Our study extends these findings, suggesting a positive association between maternal Hg levels and child risk of OWO from preschool age to school age to adolescence. These results were consistent across an array of sensitivity analyses. Our findings provide new evidence that in utero Hg exposure may play a role in the development of childhood OWO.

The underlying mechanism for the association between in utero Hg exposure and risk of child OWO is not clear. One potential mechanism is via MeHg-induced oxidative stress and inflammation. Experimental studies have demonstrated that MeHg exposure induces oxidative stress and systemic inflammation [26], which, in turn, can cause disturbances in glucose metabolism and lipid peroxidation [27]. Consistently, a human study showed that MeHg exposure was associated with reduced activity of Paraoxonase 1 (PON1) [28], an enzyme that inhibits systemic oxidative stress and guards against atherosclerosis and obesity [29].

Nutrition has been proposed as a safe, simple, and inexpensive method to mitigate the detrimental effects of exposure to environmental toxicants [30, 31]. One of the nutrients that might reduce the adverse impacts of chemicals on the fetus is folate [18]. However, the literature is limited on the relationship between folate status and Hg toxicity. Previous studies showed that folate and vitamin B12 deficiency magnify the adverse effects of Hg [32] and that folate may play a role in alleviating Hg toxicity [19]. Our findings reiterate the potential importance of optimal maternal folate status in the setting of Hg exposure.

Our study has important public health implications. MeHg is a major contaminant in some fish and seafoods. The overall health impact of fish consumption may reflect both the beneficial effects of nutrients, such as omega-3 long-chain fatty acids, and the detrimental effects of contaminants, such as Hg, found in fish. The results of the current study emphasize the need to carefully weigh the nutritional benefits of fish consumption with the risks of increased exposure to Hg during pregnancy to reduce the risk of childhood OWO. Our study findings raise the prospect to screen for Hg and folate and to optimize maternal folate status during gestation, especially among mothers with OWO and/or diabetes.

Our study has a couple of strengths. We measured child BMI longitudinally, which enabled us to define OWO over 15 years of follow-up and explore temporal characteristics of the association between in utero Hg exposure and OWO risk. In addition, we assessed in utero Hg exposure in maternal RBCs 1–3 days after delivery, reflecting third trimester exposure and free from hemodilution. However, several potential limitations of the study need to be noted. We measured total Hg, not MeHg, though Hg concentrations in RBCs are the best biomarker of MeHg exposure insofar as ∼ 80% of MeHg is stored in red blood cells [2]. Second, we did not measure child Hg exposure during childhood. Third, although we controlled for many potential confounders, such as maternal age, education, race/ethnicity, parity, smoking, and other pertinent covariates in the models, we could not completely eliminate the potential for residual confounding due to unknown or uncontrolled confounders. Finally, the fact that our study population was dominantly urban, low-income Blacks might limit the generalizability of our findings.

## Conclusion

In this large, long-term prospective birth cohort study of a US urban low-income population, there was a significant dose-response relationship between in utero Hg exposure and risk of child OWO. Maternal pre-pregnancy OWO and/or diabetes enhanced the Hg-child OWO associations, while adequate maternal folate status mitigated the associations. These findings, if further confirmed, underscore the need to screen for Hg and folate and to optimize maternal folate status during gestation, especially among mothers with OWO and diabetes.

## Supplementary information

**Additional file 1: Figure S1.** Flowchart of study population. **Figure S2.** The distribution of maternal RBC-mercury stratified by maternal race and prepregnancy overweight or obesity (OWO) and/or diabetes (DM) status. **Figure S3.** The relationship between maternal plasma folate and RBC-Hg concentrations. **Table S1.** Comparison of pre- and peri-natal characteristics between total enrolled sample, follow-up sample, included sample of this analysis and subset with folate data. **Table S2.** Individual and combined effects of maternal OWO and/or DM and mercury and child risk of OWO, with additional adjustment for frequency of fish consumption. **Table S3.** Individual and combined effects of maternal OWO and/or DM and mercury on child risk of OWO, with additional adjustment for maternal selenium level. **Table S4.** Individual and combined effects of maternal OWO and/or DM and mercury on child risk of OWO, with additional adjustment for maternal lead level. **Table S5.** Individual and combined effects of maternal OWO and/or DM and mercury on child OWO among fish consumers only (*n*=1109). **Table S6.** Individual and combined effects of maternal OWO and/or DM and mercury on child OWO among term births only (*n*=1094). **Table S7.** Individual and combined effects of maternal OWO and/or DM and mercury on child OWO among black children (*n*=967). **Table S8.** Individual and combined effects of maternal OWO and/or DM and mercury on child OWO among boys (*n*=722). **Table S9.** Individual and combined effects of maternal OWO and/or DM and mercury on child OWO among girls (*n*=720). **Table S10.** Individual and combined effects of maternal OWO and/or DM and mercury on child OWO among breastfed children only (n=1094). **Table S11.** Individual and combined effects of maternal OWO and/or DM and mercury on child BMI z-scores.

## Data Availability

The datasets used and/or analyzed during the current study are available from the corresponding author on reasonable request.

## Abbreviations

AGA: Appropriate for gestational age
BBC: The Boston Birth Cohort
BMI: Body mass index
CI: Confidence interval
CV: Coefficient variation
HELLP: Hemolysis, elevated liver enzymes, and low platelets syndrome
Hg: Mercury
IQR: Interquartile range
LGA: Large for gestational age
LOD: The limit of detection
MeHg: Methylmercury
OWO: Overweight or obesity
RR: Relative risk
SD: Standard deviation
SGA: Small for gestational age
