# Supplementary material for: In utero exposure to mercury and childhood overweight or obesity: counteracting effect of maternal folate status
Source: BMC Med. 2019 Nov 28;17:216. doi: 10.1186/s12916-019-1442-2 (PMC6882077; doi:10.1186/s12916-019-1442-2)
Supplement: Supplementary file 1 — Additional file 1: Figure S1. Flowchart of study population. Figure S2. The distribution of maternal RBC-mercury stratified by maternal race and prepregnancy overweight or obesity (OWO) and/or diabetes (DM) status. Figure S3. The relationship between maternal plasma folate and RBC-Hg concentrations. Table S1. Comparison of pre- and peri-natal characteristics between total enrolled sample, follow-up sample, included sample of this analysis and subset with folate data. Table S2. Individual and combined effects of maternal OWO and/or DM and mercury and child risk of OWO, with additional adjustment for frequency of fish consumption. Table S3. Individual and combined effects of maternal OWO and/or DM and mercury on child risk of OWO, with additional adjustment for maternal selenium level. Table S4. Individual and combined effects of maternal OWO and/or DM and mercury on child risk of OWO, with additional adjustment for maternal lead level. Table S5. Individual and combined effects of maternal OWO and/or DM and mercury on child OWO among fish consumers only (n=1109). Table S6. Individual and combined effects of maternal OWO and/or DM and mercury on child OWO among term births only (n=1094). Table S7. Individual and combined effects of maternal OWO and/or DM and mercury on child OWO among black children (n=967). Table S8. Individual and combined effects of maternal OWO and/or DM and mercury on child OWO among boys (n=722). Table S9. Individual and combined effects of maternal OWO and/or DM and mercury on child OWO among girls (n=720). Table S10. Individual and combined effects of maternal OWO and/or DM and mercury on child OWO among breastfed children only (n=1094). Table S11. Individual and combined effects of maternal OWO and/or DM and mercury on child BMI z-scores. [file 12916_2019_1442_MOESM1_ESM.docx]

**Additional file 1:**

SUPPLEMENTAL FIGURES AND TABLES

**Table of Contents**

| **ITEM** | **PAGE** |
| --- | --- |
| **Supplemental Figure** |  |
| Figure S1. Flowchart of study population | 2 |
| Figure S2. The distribution of maternal RBC-mercury stratified by maternal race and prepregnancy overweight or obesity (OWO) and/or diabetes (DM) status. | 3 |
| Figure S3. The relationship between maternal plasma folate and RBC-Hg concentrations. | 4 |
|  |  |
| **Supplemental Tables** |  |
| Table S1. Comparison of pre- and peri-natal characteristics between total enrolled sample, follow-up sample, included sample of this analysis and subset with folate data. | 5 |
| Table S2. Individual and combined effects of maternal OWO and/or DM and mercury and child risk of OWO, with additional adjustment for frequency of fish consumption. | 6 |
| Table S3. Individual and combined effects of maternal OWO and/or DM and mercury on child risk of OWO, with additional adjustment for maternal selenium level. | 7 |
| Table S4. Individual and combined effects of maternal OWO and/or DM and mercury on child risk of OWO, with additional adjustment for maternal lead level. | 8 |
| Table S5. Individual and combined effects of maternal OWO and/or DM and mercury on child OWO among fish consumers only (n=1109). | 9 |
| Table S6. Individual and combined effects of maternal OWO and/or DM and mercury on child OWO among term births only (n=1094). | 10 |
| Table S7. Individual and combined effects of maternal OWO and/or DM and mercury on child OWO among black children (n=967). | 11 |
| Table S8. Individual and combined effects of maternal OWO and/or DM and mercury on child OWO among boys (n=722). | 12 |
| Table S9. Individual and combined effects of maternal OWO and/or DM and mercury on child OWO among girls (n=720). | 13 |
| Table S10. Individual and combined effects of maternal OWO and/or DM and mercury on child OWO among breastfed children only (n=1094). | 14 |
|  |  |
| Table S11. Individual and combined effects of maternal OWO and/or DM and mercury on child BMI z-scores. | 15 |

Figure S1. Flowchart of study population


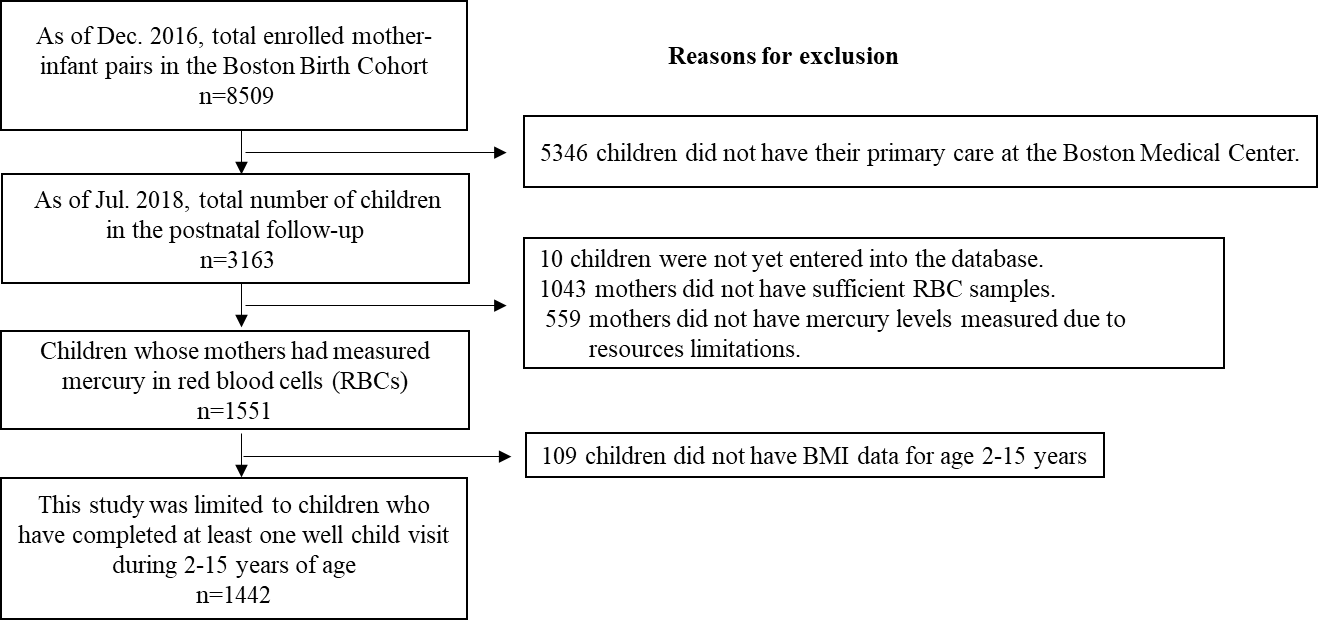


Figure S2. The distribution of maternal RBC-mercury stratified by maternal race and OWO/DM status

| A  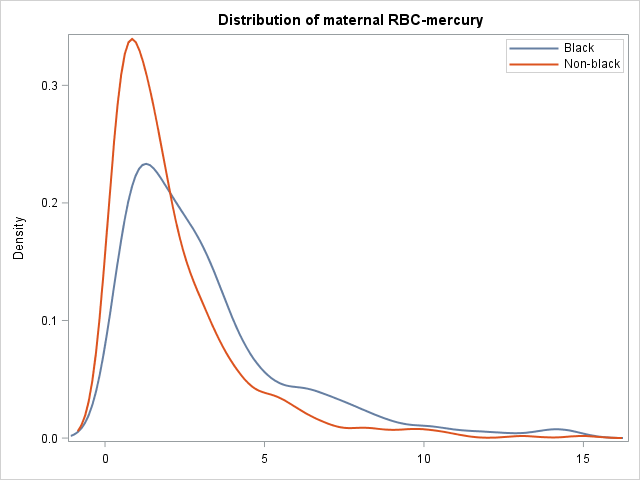 |
| --- |
| B  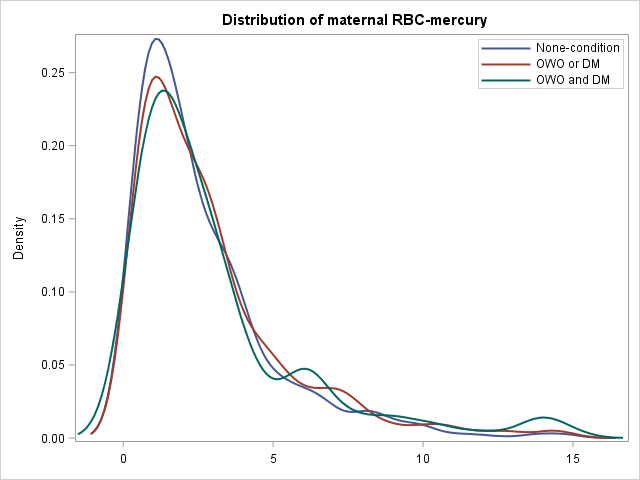 |

Panel A display the distribution of maternal RBC-mercury stratified by race; Panel B displays the distribution of maternal RBC mercury stratified by maternal OWO/DM status. The plots were truncated at 15 µg/L (n=28 for RBC-Hg>15 µg/L); OWO, overweight or obesity; DM, diabetes.

Figure S3. The relationship between maternal plasma folate and RBC-Hg concentrations.


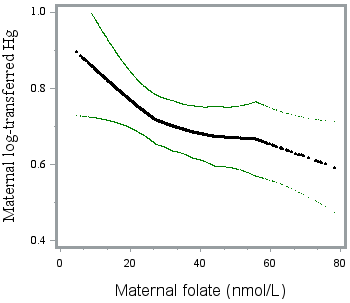


Table S1. Comparison of pre- and peri-natal characteristics between total enrolled sample, follow-up sample, included sample of this analysis and subset with folate data.

|  | Total sample in BBC | Total follow-up sample | Included in this study | Subgroup with folate data |
| --- | --- | --- | --- | --- |
| N | 8509 | 3153 | 1442 | 1209 |
| **Maternal characteristic** |  |  |  |  |
| Maternal age (years) | 28.2±6.5 | 28.5±6.5 | 28.6±6.5 | 28.5±6.5 |
| Race |  |  |  |  |
| Black | 4393(51.6) | 2011(63.8) | 967(67.1) | 834(69.0) |
| Non-black | 4116(48.4) | 1142(36.2) | 475(32.9) | 375(31.0) |
| Education |  |  |  |  |
| High school and below | 5466(64.2) | 2018(64.0) | 936(64.9) | 801(66.3) |
| College and above | 2944(34.6) | 1118(35.5) | 506(35.1) | 408(33.7) |
| Unknown | 99(1.2) | 17(0.5) | 0(0.0) | 0(0.0) |
| Smoking |  |  |  |  |
| Not smoker | 6815(80.1) | 2558(81.1) | 1192(82.7) | 1002(82.9) |
| Smoker | 1637(19.2) | 580(18.4) | 250(17.3) | 207(17.1) |
| Unknown | 57(0.7) | 15(0.5) | 0(0.0) | 0(0.0) |
| Parity |  |  |  |  |
| Nulliparous | 3663(43.1) | 1345(42.7) | 602(41.7) | 495(40.9) |
| Multiparous | 4846(56.9) | 1808(57.3) | 840(58.3) | 714(59.1) |
| Unknown |  |  |  |  |
| Prepregnancy BMI (kg/m^2^) | 26.1±6.3^e^ | 26.7±6.7^i^ | 26.8±6.7 | 26.8±6.6 |
| Overweight or obesity | 3984(48.2)^e^ | 1621(51.9)^i^ | 761(52.8) | 644(53.3) |
| Diabetes | 883(10.4)^f^ | 388(12.3) | 184(12.8) | 157(13.0) |
| Hypertensive disorder | 1094(12.9)^g^ | 482(15.3)^j^ | 216(15.0) | 177(14.6) |
| Plasma folate (nmol/L)^a^ | 30.0(19.8-43.8)^b^ | 30.6(20.4-44.3)^c^ | 32.2(22.1-44.4)^d^ | 32.2(22.1-44.4)^e^ |
| Fish consumption (serving/wk) during 3^rd^ trimester |  |  |  |  |
| 0 | 1681(19.7)^h^ | 630(20.0)^k^ | 333(23.1) | 281(23.3) |
| 1-2 | 5714(67.2) | 2088(66.3) | 921(63.9) | 768(63.5) |
| ≥3 | 1112(13.1) | 433(13.7) | 188(13.0) | 160(13.2) |
| **Child characteristic** |  |  |  |  |
| Birthweight (g) | 2957±770 | 2903±817 | 2981±789 | 2968±791 |
| Gestational age (g) | 37.9±3.2 | 37.7±3.5 | 38.0±3.3 | 37.9±3.3 |
| Preterm birth | 2316(27.2) | 905(28.7) | 348(24.1) | 296(24.5) |
| Fetal growth pattern |  |  |  |  |
| AGA | 6647(78.1) | 2456(77.9) | 1136(78.8) | 953(78.8) |
| SGA | 1037(12.2) | 381(12.1) | 151(10.5) | 125(10.3) |
| LGA | 825(9.7) | 316(10.0) | 155(10.7) | 131(10.9) |

BBC, the Boston Birth Cohort; AGA, appropriate for gestational age; SGA, small for gestational age; LGA, large for gestational age. ^a^Median(IQR); ^b^N=2598; ^c^N=2070; ^d^N=1209; ^e^N=8275; ^f^N=8500; ^g^N=8493; ^h^N=8507; ^i^N=3122; ^j^N=3151; ^k^N=3151.

Table S2. Individual and combined effects of maternal OWO and/or DM and mercury and child risk of overweight or obesity, with additional adjustment for frequency of fish consumption

| Maternal | Mercury |  |  |  | Model 1 |  |  |  | Model 2 |  |
| --- | --- | --- | --- | --- | --- | --- | --- | --- | --- | --- |
| OWO/DM | quartile | n | Case,n(%) | RR | 95%CI | p |  | RR | 95%CI | p |
|  | Q1-Q3 | 1080 | 438(40.6) | 1.00 |  |  |  | 1.00 |  |  |
|  | Q4 | 362 | 174(48.1) | 1.18 | 1.04-1.35 | 0.012 |  | 1.19 | 1.04-1.36 | 0.012 |
| **OWO** |  |  |  |  |  |  |  |  |  |  |
| No | Q1-Q3 | 516 | 152(29.5) | 1.00 |  |  |  | 1.00 |  |  |
|  | Q4 | 165 | 61(37.0) | 1.28 | 1.01-1.63 | 0.045 |  | 1.28 | 1.01-1.64 | 0.044 |
| Yes | Q1-Q3 | 564 | 286(50.7) | 1.63 | 1.39-1.92 | <0.001 |  | 1.63 | 1.39-1.91 | <0.001 |
|  | Q4 | 197 | 113(57.4) | 1.87 | 1.55-2.25 | <0.001 |  | 1.88 | 1.55-2.27 | <0.001 |
| **DM** |  |  |  |  |  |  |  |  |  |  |
| No | Q1-Q3 | 946 | 362(38.3) | 1.00 |  |  |  | 1.00 |  |  |
|  | Q4 | 312 | 144(46.2) | 1.20 | 1.03-1.39 | 0.015 |  | 1.21 | 1.04-1.40 | 0.015 |
| Yes | Q1-Q3 | 134 | 76(56.7) | 1.31 | 1.10-1.55 | 0.002 |  | 1.31 | 1.10-1.55 | 0.003 |
|  | Q4 | 50 | 30(60.0) | 1.45 | 1.15-1.83 | 0.002 |  | 1.46 | 1.15-1.84 | 0.002 |
| **OWO/DM** |  |  |  |  |  |  |  |  |  |  |
| None | Q1-Q3 | 486 | 140(28.8) | 1.00 |  |  |  | 1.00 |  |  |
|  | Q4 | 151 | 54(35.8) | 1.27 | 0.98-1.64 | 0.071 |  | 1.27 | 0.98-1.65 | 0.071 |
| Any | Q1-Q3 | 490 | 234(47.8) | 1.61 | 1.35-1.90 | <0.001 |  | 1.60 | 1.35-1.90 | <0.001 |
|  | Q4 | 175 | 97(55.4) | 1.88 | 1.54-2.29 | <0.001 |  | 1.88 | 1.54-2.32 | <0.001 |
| Both | Q1-Q3 | 104 | 64(61.5) | 1.89 | 1.52-2.34 | <0.001 |  | 1.88 | 1.52-2.34 | <0.001 |
|  | Q4 | 36 | 23(63.9) | 2.06 | 1.56-2.71 | <0.001 |  | 2.06 | 1.55-2.73 | <0.001 |

OWO, overweight or obesity; DM, diabetes; RR, relative risk; Q, quartile. Mercury quartile range: Q1-Q3, 0.39-3.68 µg/L; Q4: 3.70-27.8µg/L.

Model 1: adjusted for maternal age, race, smoking, education, parity, hypertensive disorder, preterm birth, fetal growth, and breastfeeding.

Model 2: Model 1+frequency of fish consumption.

Table S3. Individual and combined effects of maternal OWO and/or DM and mercury on child risk of OWO, with additional adjustment for maternal selenium level.

| Maternal | Mercury |  |  |  | Model 1 |  |  |  | Model 2 |  |
| --- | --- | --- | --- | --- | --- | --- | --- | --- | --- | --- |
| OWO/DM | quartile | n | Case,n(%) | RR | 95%CI | p |  | OR | 95%CI | p |
|  | Q1-Q3 | 1080 | 438(40.6) | 1.00 |  |  |  | 1.00 |  |  |
|  | Q4 | 362 | 174(48.1) | 1.18 | 1.04-1.35 | 0.012 |  | 1.19 | 1.04-1.36 | 0.009 |
| **OWO** |  |  |  |  |  |  |  |  |  |  |
| No | Q1-Q3 | 516 | 152(29.5) | 1.00 |  |  |  | 1.00 |  |  |
|  | Q4 | 165 | 61(37.0) | 1.28 | 1.01-1.63 | 0.045 |  | 1.30 | 1.02-1.65 | 0.037 |
| Yes | Q1-Q3 | 564 | 286(50.7) | 1.63 | 1.39-1.92 | <0.001 |  | 1.64 | 1.39-1.92 | <0.001 |
|  | Q4 | 197 | 113(57.4) | 1.87 | 1.55-2.25 | <0.001 |  | 1.90 | 1.57-2.30 | <0.001 |
| **DM** |  |  |  |  |  |  |  |  |  |  |
| No | Q1-Q3 | 946 | 362(38.3) | 1.00 |  |  |  | 1.00 |  |  |
|  | Q4 | 312 | 144(46.2) | 1.20 | 1.03-1.39 | 0.015 |  | 1.21 | 1.04-1.41 | 0.011 |
| Yes | Q1-Q3 | 134 | 76(56.7) | 1.31 | 1.10-1.55 | 0.002 |  | 1.31 | 1.10-1.55 | 0.002 |
|  | Q4 | 50 | 30(60.0) | 1.45 | 1.15-1.83 | 0.002 |  | 1.47 | 1.17-1.86 | 0.001 |
| **OWO/DM** |  |  |  |  |  |  |  |  |  |  |
| None | Q1-Q3 | 486 | 140(28.8) | 1.00 |  |  |  | 1.00 |  |  |
|  | Q4 | 151 | 54(35.8) | 1.27 | 0.98-1.64 | 0.071 |  | 1.28 | 0.99-1.66 | 0.060 |
| Any | Q1-Q3 | 490 | 234(47.8) | 1.61 | 1.35-1.90 | <0.001 |  | 1.61 | 1.36-1.91 | <0.001 |
|  | Q4 | 175 | 97(55.4) | 1.88 | 1.54-2.29 | <0.001 |  | 1.91 | 1.56-2.34 | <0.001 |
| Both | Q1-Q3 | 104 | 64(61.5) | 1.89 | 1.52-2.34 | <0.001 |  | 1.89 | 1.53-2.34 | <0.001 |
|  | Q4 | 36 | 23(63.9) | 2.06 | 1.56-2.71 | <0.001 |  | 2.10 | 1.59-2.77 | <0.001 |

OWO, overweight or obesity; DM, diabetes; RR, relative risk; Q, quartile. Mercury quartile range: Q1-Q3: 0.39-3.68 µg/L; Q4: 3.70-27.8µg/L.

Model 1: adjusted for maternal age, race, smoking, education, parity, hypertensive disorder, preterm birth, fetal growth, and breastfeeding.

Model 2: Model 1+maternal selenium concentrations.

Table S4. Individual and combined effects of maternal OWO and/or DM and mercury on child risk of OWO, with additional adjustment for maternal lead level.

| Maternal | Mercury |  |  |  | Model 1 |  |  |  | Model 2 |  |
| --- | --- | --- | --- | --- | --- | --- | --- | --- | --- | --- |
| OWO/DM | quartile | n | Case,n(%) | RR | 95%CI | p |  | RR | 95%CI | p |
|  | Q1-Q3 | 1080 | 438(40.6) | 1.00 |  |  |  | 1.00 |  |  |
|  | Q4 | 362 | 174(48.1) | 1.18 | 1.04-1.35 | 0.012 |  | 1.16 | 1.02-1.33 | 0.024 |
| **OWO** |  |  |  |  |  |  |  |  |  |  |
| No | Q1-Q3 | 516 | 152(29.5) | 1.00 |  |  |  | 1.00 |  |  |
|  | Q4 | 165 | 61(37.0) | 1.28 | 1.01-1.63 | 0.045 |  | 1.26 | 0.99-1.60 | 0.065 |
| Yes | Q1-Q3 | 564 | 286(50.7) | 1.63 | 1.39-1.92 | <0.001 |  | 1.63 | 1.39-1.91 | <0.001 |
|  | Q4 | 197 | 113(57.4) | 1.87 | 1.55-2.25 | <0.001 |  | 1.84 | 1.52-2.22 | <0.001 |
| **DM** |  |  |  |  |  |  |  |  |  |  |
| No | Q1-Q3 | 946 | 362(38.3) | 1.00 |  |  |  | 1.00 |  |  |
|  | Q4 | 312 | 144(46.2) | 1.20 | 1.03-1.39 | 0.015 |  | 1.19 | 1.02-1.38 | 0.024 |
| Yes | Q1-Q3 | 134 | 76(56.7) | 1.31 | 1.10-1.55 | 0.002 |  | 1.31 | 1.10-1.55 | 0.002 |
|  | Q4 | 50 | 30(60.0) | 1.45 | 1.15-1.83 | 0.002 |  | 1.42 | 1.12-1.79 | 0.004 |
| **OWO/DM** |  |  |  |  |  |  |  |  |  |  |
| None | Q1-Q3 | 486 | 140(28.8) | 1.00 |  |  |  | 1.00 |  |  |
|  | Q4 | 151 | 54(35.8) | 1.27 | 0.98-1.64 | 0.071 |  | 1.25 | 0.96-1.62 | 0.092 |
| Any | Q1-Q3 | 490 | 234(47.8) | 1.61 | 1.35-1.90 | <0.001 |  | 1.61 | 1.36-1.90 | <0.001 |
|  | Q4 | 175 | 97(55.4) | 1.88 | 1.54-2.29 | <0.001 |  | 1.85 | 1.52-2.27 | <0.001 |
| Both | Q1-Q3 | 104 | 64(61.5) | 1.89 | 1.52-2.34 | <0.001 |  | 1.88 | 1.51-2.33 | <0.001 |
|  | Q4 | 36 | 23(63.9) | 2.06 | 1.56-2.71 | <0.001 |  | 2.01 | 1.52-2.66 | <0.001 |

OWO, overweight or obesity; DM, diabetes; RR, relative risk; Q, quartile. Mercury quartile range: Q1-Q3, 0.39-3.68 µg/L; Q4: 3.70-27.8µg/L.

Model 1: adjusted for maternal age, race, smoking, education, parity, hypertensive disorder, preterm birth, fetal growth, and breastfeeding.

Model 2: Model 1+ maternal lead level.

Table S5. Individual and combined effects of maternal OWO and/or DM and mercury on child OWO among fish consumers only (n=1109)

| Maternal | Mercury |  |  |  | Crude |  |  |  | Adjusted |  |
| --- | --- | --- | --- | --- | --- | --- | --- | --- | --- | --- |
| OWO/DM | quartile | n | Case,n(%) | RR | 95%CI | p |  | RR | 95%CI | p |
|  | Q1-Q3 | 782 | 315(40.3) | 1.00 |  |  |  | 1.00 |  |  |
|  | Q4 | 327 | 158(48.3) | 1.20 | 1.04-1.38 | 0.011 |  | 1.19 | 1.03-1.37 | 0.015 |
| **OWO** |  |  |  |  |  |  |  |  |  |  |
| No | Q1-Q3 | 371 | 114(30.7) | 1.00 |  |  |  | 1.00 |  |  |
|  | Q4 | 143 | 53(37.1) | 1.21 | 0.93-1.57 | 0.162 |  | 1.22 | 0.94-1.60 | 0.135 |
| Yes | Q1-Q3 | 411 | 201(48.9) | 1.59 | 1.33-1.91 | <0.001 |  | 1.51 | 1.25-1.81 | <0.001 |
|  | Q4 | 184 | 105(57.1) | 1.86 | 1.52-2.26 | <0.001 |  | 1.76 | 1.43-2.17 | <0.001 |
| **DM** |  |  |  |  |  |  |  |  |  |  |
| No | Q1-Q3 | 685 | 260(38.0) | 1.00 |  |  |  | 1.00 |  |  |
|  | Q4 | 281 | 129(45.9) | 1.21 | 1.03-1.42 | 0.019 |  | 1.20 | 1.02-1.41 | 0.025 |
| Yes | Q1-Q3 | 97 | 55(56.7) | 1.49 | 1.22-1.82 | <0.001 |  | 1.32 | 1.08-1.62 | 0.007 |
|  | Q4 | 46 | 29(63.0) | 1.66 | 1.31-2.11 | <0.001 |  | 1.52 | 1.21-1.93 | <0.001 |
| **OWO/DM** |  |  |  |  |  |  |  |  |  |  |
| None | Q1-Q3 | 350 | 104(29.7) | 1.00 |  |  |  | 1.00 |  |  |
|  | Q4 | 131 | 47(35.9) | 1.21 | 0.91-1.60 | 0.187 |  | 1.23 | 0.93-1.64 | 0.149 |
| Any | Q1-Q3 | 356 | 166(46.6) | 1.57 | 1.29-1.91 | <0.001 |  | 1.52 | 1.25-1.86 | <0.001 |
|  | Q4 | 162 | 88(54.3) | 1.83 | 1.48-2.26 | <0.001 |  | 1.77 | 1.42-2.21 | <0.001 |
| Both | Q1-Q3 | 76 | 45(59.2) | 1.99 | 1.56-2.55 | <0.001 |  | 1.75 | 1.35-2.27 | <0.001 |
|  | Q4 | 34 | 23(67.7) | 2.28 | 1.72-3.02 | <0.001 |  | 2.09 | 1.57-2.80 | <0.001 |

OWO, overweight or obesity; DM, diabetes; RR, relative risk; Q, quartile. Mercury quartile range: Q1-Q3, 0.39-3.68 µg/L; Q4: 3.70-27.8µg/L.

Adjusted for maternal age, race, smoking, education, parity, hypertensive disorder, preterm birth, fetal growth, and breastfeeding.

Table S6. Individual and combined effects of maternal OWO and/or DM and mercury on child OWO among term births only (n=1094)

| Maternal | Mercury |  |  |  | Crude |  |  |  | Adjusted |  |
| --- | --- | --- | --- | --- | --- | --- | --- | --- | --- | --- |
| OWO/DM | quartile | n | Case,n(%) | RR | 95%CI | p |  | RR | 95%CI | p |
|  | Q1-Q3 | 817 | 333(40.8) | 1.00 |  |  |  | 1.00 |  |  |
|  | Q4 | 277 | 134(48.4) | 1.19 | 1.02-1.37 | 0.022 |  | 1.15 | 0.99-1.34 | 0.064 |
| **OWO** |  |  |  |  |  |  |  |  |  |  |
| No | Q1-Q3 | 401 | 117(29.2) | 1.00 |  |  |  | 1.00 |  |  |
|  | Q4 | 125 | 46(36.8) | 1.26 | 0.96-1.66 | 0.099 |  | 1.25 | 0.95-1.65 | 0.116 |
| Yes | Q1-Q3 | 416 | 216(51.9) | 1.78 | 1.49-2.13 | <0.001 |  | 1.65 | 1.37-1.99 | <0.001 |
|  | Q4 | 152 | 88(57.9) | 1.98 | 1.62-2.43 | <0.001 |  | 1.82 | 1.47-2.25 | <0.001 |
| **DM** |  |  |  |  |  |  |  |  |  |  |
| No | Q1-Q3 | 725 | 274(37.8) | 1.00 |  |  |  | 1.00 |  |  |
|  | Q4 | 243 | 112(46.1) | 1.22 | 1.03-1.44 | 0.018 |  | 1.19 | 1.00-1.40 | 0.048 |
| Yes | Q1-Q3 | 92 | 59(64.1) | 1.70 | 1.42-2.03 | <0.001 |  | 1.49 | 1.24-1.80 | <0.001 |
|  | Q4 | 34 | 22(64.7) | 1.71 | 1.31-2.13 | <0.001 |  | 1.53 | 1.17-1.99 | 0.002 |
| **OWO/DM** |  |  |  |  |  |  |  |  |  |  |
| None | Q1-Q3 | 378 | 106(28.0) | 1.00 |  |  |  | 1.00 |  |  |
|  | Q4 | 115 | 41(35.7) | 1.27 | 0.95-1.71 | 0.109 |  | 1.27 | 0.94-1.70 | 0.119 |
| Any | Q1-Q3 | 370 | 179(48.4) | 1.73 | 1.42-2.09 | <0.001 |  | 1.64 | 1.35-2.00 | <0.001 |
|  | Q4 | 138 | 76(55.1) | 1.96 | 1.57-2.45 | <0.001 |  | 1.84 | 1.47-2.32 | <0.001 |
| Both | Q1-Q3 | 69 | 48(69.6) | 2.48 | 1.98-3.11 | <0.001 |  | 2.16 | 1.69-2.73 | <0.001 |
|  | Q4 | 24 | 17(70.8) | 2.53 | 1.87-3.42 | <0.001 |  | 2.24 | 1.64-3.05 | <0.001 |

OWO, overweight or obesity; DM, diabetes; RR, relative risk; Q, quartile. Mercury quartile range: Q1-Q3, 0.39-3.68 µg/L; Q4: 3.70-27.8µg/L.

Adjusted for maternal age, race, smoking, education, parity, hypertensive disorder, fetal growth, and breastfeeding.

Table S7. Individual and combined effects of maternal OWO and/or DM and mercury on child OWO among black children (n=967)

| Maternal | Mercury |  |  |  | Crude |  |  |  | Adjusted |  |
| --- | --- | --- | --- | --- | --- | --- | --- | --- | --- | --- |
| OWO/DM | quartile | n | Case,n(%) | RR | 95%CI | p |  | RR | 95%CI | p |
|  | Q1-Q3 | 682 | 281(41.2) | 1.00 |  |  |  | 1.00 |  |  |
|  | Q4 | 285 | 131(46.0) | 1.12 | 0.96-1.30 | 0.165 |  | 1.11 | 0.95-1.30 | 0.180 |
| **OWO** |  |  |  |  |  |  |  |  |  |  |
| No | Q1-Q3 | 306 | 89(29.1) | 1.00 |  |  |  | 1.00 |  |  |
|  | Q4 | 121 | 41(33.9) | 1.16 | 0.86-1.58 | 0.325 |  | 1.16 | 0.86-1.58 | 0.333 |
| Yes | Q1-Q3 | 376 | 192(51.1) | 1.76 | 1.44-2.15 | <0.001 |  | 1.65 | 1.34-2.02 | <0.001 |
|  | Q4 | 164 | 90(54.9) | 1.89 | 1.51-2.36 | <0.001 |  | 1.79 | 1.42-2.25 | <0.001 |
| **DM** |  |  |  |  |  |  |  |  |  |  |
| No | Q1-Q3 | 598 | 233(39.0) | 1.00 |  |  |  | 1.00 |  |  |
|  | Q4 | 244 | 108(44.3) | 1.14 | 0.96-1.35 | 0.148 |  | 1.13 | 0.95-1.35 | 0.168 |
| Yes | Q1-Q3 | 84 | 48(57.1) | 1.47 | 1.19-1.81 | <0.001 |  | 1.32 | 1.07-1.63 | 0.010 |
|  | Q4 | 41 | 23(56.1) | 1.44 | 1.08-1.92 | 0.013 |  | 1.36 | 1.03-1.80 | 0.032 |
| **OWO/DM** |  |  |  |  |  |  |  |  |  |  |
| None | Q1-Q3 | 289 | 83(28.7) | 1.00 |  |  |  | 1.00 |  |  |
|  | Q4 | 112 | 37(33.0) | 1.15 | 0.84-1.58 | 0.391 |  | 1.15 | 0.83-1.59 | 0.397 |
| Any | Q1-Q3 | 326 | 156(47.9) | 1.67 | 1.35-2.06 | <0.001 |  | 1.59 | 1.28-1.97 | <0.001 |
|  | Q4 | 141 | 75(53.2) | 1.85 | 1.46-2.35 | <0.001 |  | 1.78 | 1.39-2.27 | <0.001 |
| Both | Q1-Q3 | 67 | 42(62.7) | 2.18 | 1.68-2.83 | <0.001 |  | 1.97 | 1.51-2.56 | <0.001 |
|  | Q4 | 32 | 19(59.4) | 2.07 | 1.47-2.90 | <0.001 |  | 1.94 | 1.38-2.71 | <0.001 |

OWO, overweight or obesity; DM, diabetes; RR, relative risk; Q, quartile. Mercury quartile range: Q1-Q3, 0.39-3.68 µg/L; Q4: 3.70-27.8µg/L.

Adjusted for maternal age, smoking, education, parity, hypertensive disorder, preterm birth, fetal growth, and breastfeeding.

Table S8. Individual and combined effects of maternal OWO and/or DM and mercury on child OWO among boys (n=722)

| Maternal | Mercury |  |  |  | Crude |  |  |  | Adjusted |  |
| --- | --- | --- | --- | --- | --- | --- | --- | --- | --- | --- |
| OWO/DM | quartile | n | Case,n(%) | RR | 95%CI | p |  | RR | 95%CI | p |
|  | Q1-Q3 | 566 | 235(41.5) | 1.00 |  |  |  | 1.00 |  |  |
|  | Q4 | 156 | 75(48.1) | 1.16 | 0.96-1.40 | 0.131 |  | 1.19 | 0.99-1.44 | 0.070 |
| **OWO** |  |  |  |  |  |  |  |  |  |  |
| No | Q1-Q3 | 271 | 87(32.1) | 1.00 |  |  |  | 1.00 |  |  |
|  | Q4 | 65 | 22(33.9) | 1.05 | 0.72-1.54 | 0.786 |  | 1.08 | 0.74-1.58 | 0.703 |
| Yes | Q1-Q3 | 295 | 148(50.2) | 1.56 | 1.27-1.92 | <0.001 |  | 1.45 | 1.17-1.79 | <0.001 |
|  | Q4 | 91 | 53(58.2) | 1.81 | 1.42-2.32 | <0.001 |  | 1.75 | 1.37-2.24 | <0.001 |
| **DM** |  |  |  |  |  |  |  |  |  |  |
| No | Q1-Q3 | 485 | 189(39.0) | 1.00 |  |  |  | 1.00 |  |  |
|  | Q4 | 135 | 66(48.9) | 1.25 | 1.02-1.54 | 0.030 |  | 1.28 | 1.04-1.57 | 0.020 |
| Yes | Q1-Q3 | 81 | 46(56.8) | 1.46 | 1.17-1.82 | <0.001 |  | 1.27 | 1.01-1.60 | 0.045 |
|  | Q4 | 21 | 9(42.9) | 1.10 | 0.66-2.00 | 0.713 |  | 1.02 | 0.65-1.62 | 0.929 |
| **OWO/DM** |  |  |  |  |  |  |  |  |  |  |
| None | Q1-Q3 | 249 | 76(30.5) | 1.00 |  |  |  | 1.00 |  |  |
|  | Q4 | 59 | 21(35.6) | 1.17 | 0.79-1.72 | 0.441 |  | 1.19 | 0.80-1.76 | 0.393 |
| Any | Q1-Q3 | 258 | 124(48.1) | 1.57 | 1.26-1.97 | <0.001 |  | 1.51 | 1.20-1.89 | <0.001 |
|  | Q4 | 82 | 46(56.1) | 1.84 | 1.41-2.40 | <0.001 |  | 1.83 | 1.40-2.41 | <0.001 |
| Both | Q1-Q3 | 59 | 35(59.3) | 1.94 | 1.47-2.58 | <0.001 |  | 1.63 | 1.20-2.21 | 0.002 |
|  | Q4 | 15 | 8(53.3) | 1.75 | 1.05-2.91 | 0.032 |  | 1.52 | 0.95-2.44 | 0.081 |

OWO, overweight or obesity; DM, diabetes; RR, relative risk; Q, quartile. Mercury quartile range: Q1-Q3, 0.39-3.68 µg/L; Q4: 3.70-27.8µg/L.

Adjusted for maternal age, race, smoking, education, parity, hypertensive disorder, preterm birth, fetal growth, and breastfeeding.

Table S9. Individual and combined effects of maternal OWO and/or DM and mercury on child OWO among girls (n=720)

| Maternal | Mercury |  |  |  | Crude |  |  |  | Adjusted |  |
| --- | --- | --- | --- | --- | --- | --- | --- | --- | --- | --- |
| OWO/DM | quartile | n | Case,n(%) | RR | 95%CI | p |  | RR | 95%CI | p |
|  | Q1-Q3 | 514 | 203(39.5) | 1.00 |  |  |  | 1.00 |  |  |
|  | Q4 | 206 | 99(48.1) | 1.22 | 1.02-1.45 | 0.031 |  | 1.19 | 0.99-1.43 | 0.060 |
| **OWO** |  |  |  |  |  |  |  |  |  |  |
| No | Q1-Q3 | 245 | 65(26.5) | 1.00 |  |  |  | 1.00 |  |  |
|  | Q4 | 100 | 39(39.0) | 1.47 | 1.07-2.03 | 0.019 |  | 1.50 | 1.08-2.07 | 0.015 |
| Yes | Q1-Q3 | 269 | 138(51.3) | 1.93 | 1.52-2.45 | <0.001 |  | 1.87 | 1.46-2.39 | <0.001 |
|  | Q4 | 106 | 60(56.6) | 2.13 | 1.63-2.79 | <0.001 |  | 2.07 | 1.56-2.76 | <0.001 |
| **DM** |  |  |  |  |  |  |  |  |  |  |
| No | Q1-Q3 | 461 | 173(37.5) | 1.00 |  |  |  | 1.00 |  |  |
|  | Q4 | 177 | 78(44.1) | 1.17 | 0.96-1.44 | 0.122 |  | 1.16 | 0.94-1.43 | 0.165 |
| Yes | Q1-Q3 | 53 | 30(56.6) | 1.51 | 1.16-1.96 | 0.002 |  | 1.35 | 1.04-1.77 | 0.027 |
|  | Q4 | 29 | 21(72.4) | 1.93 | 1.50-2.49 | <0.001 |  | 1.84 | 1.40-2.42 | <0.001 |
| **OWO/DM** |  |  |  |  |  |  |  |  |  |  |
| None | Q1-Q3 | 237 | 64(27.0) | 1.00 |  |  |  | 1.00 |  |  |
|  | Q4 | 92 | 33(35.9) | 1.33 | 0.94-1.87 | 0.106 |  | 1.36 | 0.96-1.93 | 0.081 |
| Any | Q1-Q3 | 232 | 110(47.4) | 1.76 | 1.37-2.25 | <0.001 |  | 1.72 | 1.33-2.22 | <0.001 |
|  | Q4 | 93 | 51(54.8) | 2.03 | 1.54-2.68 | <0.001 |  | 1.99 | 1.49-2.69 | <0.001 |
| Both | Q1-Q3 | 45 | 29(64.4) | 2.39 | 1.17-1.77 | <0.001 |  | 2.22 | 1.62-3.05 | <0.001 |
|  | Q4 | 21 | 15(71.4) | 2.65 | 1.88-3.72 | <0.001 |  | 2.64 | 1.82-3.83 | <0.001 |

OWO, overweight or obesity; DM, diabetes; RR, relative risk; Q, quartile. Mercury quartile range: Q1-Q3, 0.39-3.68 µg/L; Q4: 3.70-27.8µg/L.

Adjusted for maternal age, race, smoking, education, parity, hypertensive disorder, preterm birth, fetal growth, and breastfeeding.

Table S10. Individual and combined effects of maternal OWO and/or DM and mercury on child OWO among breastfed children only (n=1094)

| Maternal | Mercury |  |  |  | Crude |  |  |  | Adjusted |  |
| --- | --- | --- | --- | --- | --- | --- | --- | --- | --- | --- |
| OWO/DM | quartile | n | Case,n(%) | RR | 95%CI | p |  | RR | 95%CI | p |
|  | Q1-Q3 | 797 | 303(38.0) | 1.00 |  |  |  | 1.00 |  |  |
|  | Q4 | 297 | 141(47.5) | 1.24 | 1.08-1.45 | 0.004 |  | 1.23 | 1.07-1.44 | 0.006 |
| **OWO** |  |  |  |  |  |  |  |  |  |  |
| No | Q1-Q3 | 389 | 106(27.3) | 1.00 |  |  |  | 1.00 |  |  |
|  | Q4 | 138 | 48(34.8) | 1.28 | 0.96-1.69 | 0.088 |  | 1.30 | 0.97-1.73 | 0.074 |
| Yes | Q1-Q3 | 408 | 197(48.3) | 1.77 | 1.46-2.14 | <0.001 |  | 1.68 | 1.38-2.05 | <0.001 |
|  | Q4 | 159 | 93(58.5) | 2.15 | 1.74-2.64 | <0.001 |  | 2.05 | 1.64-2.55 | <0.001 |
| **DM** |  |  |  |  |  |  |  |  |  |  |
| No | Q1-Q3 | 707 | 256(36.2) | 1.00 |  |  |  | 1.00 |  |  |
|  | Q4 | 256 | 118(46.1) | 1.27 | 1.08-1.50 | 0.004 |  | 1.26 | 1.07-1.49 | 0.007 |
| Yes | Q1-Q3 | 90 | 47(52.2) | 1.44 | 1.16-1.80 | 0.001 |  | 1.29 | 1.03-1.62 | 0.027 |
|  | Q4 | 23 | 29(56.1) | 1.55 | 1.16-2.07 | 0.003 |  | 1.46 | 1.10-1.93 | 0.009 |
| **OWO/DM** |  |  |  |  |  |  |  |  |  |  |
| None | Q1-Q3 | 369 | 99(26.8) | 1.00 |  |  |  | 1.00 |  |  |
|  | Q4 | 126 | 43(34.1) | 1.27 | 0.95-1.71 | 0.110 |  | 1.30 | 0.96-1.75 | 0.092 |
| Any | Q1-Q3 | 358 | 164(45.8) | 1.71 | 1.39-2.09 | <0.001 |  | 1.64 | 1.34-2.02 | <0.001 |
|  | Q4 | 142 | 80(56.3) | 2.10 | 1.68-2.62 | <0.001 |  | 2.02 | 1.61-2.55 | <0.001 |
| Both | Q1-Q3 | 70 | 40(57.1) | 2.13 | 1.64-3.03 | <0.001 |  | 1.92 | 1.46-2.53 | <0.001 |
|  | Q4 | 29 | 18(62.1) | 2.31 | 1.66-3.22 | <0.001 |  | 2.18 | 1.57-3.04 | <0.001 |

OWO, overweight or obesity; DM, diabetes; RR, relative risk; Q, quartile. Mercury quartile range: Q1-Q3, 0.39-3.68 µg/L; Q4: 3.70-27.8µg/L.

Adjusted for maternal age, race, smoking, education, parity, hypertensive disorder, preterm birth, and fetal growth.

Table S11. Individual and combined associations of maternal mercury and pre-pregnancy OWO and/or diabetes with child BMI z-scores.

|  | Mercury |  |  | Crude | | |  | Adjusted | | |
| --- | --- | --- | --- | --- | --- | --- | --- | --- | --- | --- |
| OWO/DM | quartile | n | Mean±SD | β | se | p |  | β | se | p |
|  | Q1 | 360 | 0.61±1.18 | ref |  |  |  | ref |  |  |
|  | Q2 | 361 | 0.74±1.24 | 0·13 | 0·09 | 0·148 |  | 0·11 | 0·09 | 0·209 |
|  | Q3 | 359 | 0.68±1.25 | 0·07 | 0·09 | 0·414 |  | 0·03 | 0·09 | 0·755 |
|  | Q4 | 362 | 0.87±1.19 | 0·26 | 0·09 | 0·004 |  | 0·24 | 0·09 | 0·008 |
|  | P trend |  |  |  |  | 0·044 |  |  |  | 0·121 |
|  | Q1-Q3 | 1080 | 0.68±1.22 | ref |  |  |  | ref |  |  |
|  | Q4 | 362 | 0.87±1.19 | 0·19 | 0·07 | 0·009 |  | 0·19 | 0·07 | 0·008 |
| OWO |  |  |  |  |  |  |  |  |  |  |
| No | Q1-Q3 | 516 | 0.34±1.21 | ref |  |  |  | ref |  |  |
|  | Q4 | 165 | 0.59±1.20 | 0.25 | 0.11 | 0.018 |  | 0.27 | 0.10 | 0.011 |
| Yes | Q1-Q3 | 564 | 0.99±1.15 | 0.65 | 0.07 | <0.001 |  | 0.57 | 0.07 | <0.001 |
|  | Q4 | 197 | 1.11±1.13 | 0.77 | 0.10 | <0.001 |  | 0.70 | 0.10 | <0.001 |
| DM |  |  |  |  |  |  |  |  |  |  |
| No | Q1-Q3 | 946 | 0.61±1.22 | ref |  |  |  | ref |  |  |
|  | Q4 | 312 | 0.85±1.15 | 0.24 | 0.08 | 0.002 |  | 0.23 | 0.08 | 0.004 |
| Yes | Q1-Q3 | 134 | 1.18±1.14 | 0.57 | 0.11 | <0.001 |  | 0.40 | 0.11 | <0.001 |
|  | Q4 | 50 | 1.02±1.42 | 0.42 | 0.17 | 0.017 |  | 0.31 | 0.17 | 0.074 |
| OWO/DM |  |  |  |  |  |  |  |  |  |  |
| No | Q1-Q3 | 486 | 0.33±1.22 | ref |  |  |  | ref |  |  |
|  | Q4 | 151 | 0.58±1.17 | 0.25 | 0.11 | 0.022 |  | 0.27 | 0.11 | 0.013 |
| Any | Q1-Q3 | 490 | 0.89±1.15 | 0.56 | 0.07 | <0.001 |  | 0.51 | 0.08 | <0.001 |
|  | Q4 | 175 | 1.07±1.11 | 0.75 | 0.10 | <0.001 |  | 0.70 | 0.10 | <0.001 |
| Both | Q1-Q3 | 104 | 1.35±1.09 | 1.02 | 0.13 | <0.001 |  | 0.85 | 0.13 | <0.001 |
|  | Q4 | 36 | 1.14±1.40 | 0.81 | 0.20 | <0.001 |  | 0.69 | 0.20 | <0.001 |

OWO, overweight or obesity; DM, diabetes; Q, quartile. Mercury quartile range: Q1-Q3, 0.39-3.68 µg/L; Q4: 3.70-27.8µg/L.

Adjusted for maternal age, race, smoking, education, parity, hypertensive disorder, preterm birth, fetal growth, and breastfeeding.
